# Comprehensive phylogeny of *Konosirus punctatus* (Clupeiformes: Clupeidae) based on transcriptomic data

**DOI:** 10.1042/BSR20210455

**Published:** 2021-05-14

**Authors:** Fangrui Lou, Shengyao Qiu, Yongzheng Tang, Zhiyang Wang, Lei Wang

**Affiliations:** School of Ocean, Yantai University, Yantai 264005, China

**Keywords:** De nove assembly, Konosirus punctatus, phylogenetic analysis, Transcriptome

## Abstract

*Konosirus punctatus* is an important species for the structure of marine ecosystems. Meanwhile, it is a native species in the northwest Pacific Ocean and supports important commercial fishery. In the present study, we generated the whole transcriptome of *K. punctatus* from combined tissues (muscle, liver, gill, heart, kidney, swim bladder and sexual gland) using Illumina RNA-seq technology and a total of 46087110 clean reads were obtained, corresponding to 6531521430 nucleotides. Meanwhile, 10000 clean reads were randomly selected and compared with NT database to examine the possible contamination. Results showed that 6754 clean reads were distributed among some species closely related with *K. punctatus*, indicating no-pollution. *De novo* assembly was performed and all clean reads were assembled to produce 71610 longest unigenes with an N50 of 906 bp. Among all the unigenes, 43974 unigenes were annotated in at least one database and 3172 unigenes were annotated in all databases. All unigenes were further analyzed to predict the gene structure and we have obtained a total of 54864 coding sequences (CDSs) and 17326 simple sequence repeats (SSRs). Saturation analyses were applied to evaluate the accuracy of gene expression and we hypothesized that the detection of gene expression might be effective. Finally, single-copy orthologous genes were applied to construct the phylogenetic relationship of *K. punctatus*. Results showed that *K. punctatus* diverged from the common ancestor with *Alosa alosa*, *Alosa pseudoharengus* and *Sardina pilchardus* at approx. 61.16–92.52 MYA. The present study will provide a foundational molecular information for the biological research of *K. punctatus*.

## Introduction

*Konosirus punctatus* belongs to the family Clupeidae, which is widely distributed in the coastal waters of the northwest Pacific Ocean [1]. *K. punctatus* often swim in groups in the gulf with the depth ranging from 5 to 15 m. It can tolerate a wide range of salinity and even migrate into the freshwater [1]. *K. punctatus* usually spawns when water temperatures reach between 13.0 and 20.9°C [2]. Additionally, *K. punctatus* is an important cornerstone species of marine food webs, since it primarily feeds on phytoplankton [3]. Therefore, *K. punctatus* is very important for the structure of marine ecosystems. Previous studies also showed that it is a native species in the northwest Pacific Ocean and supports important commercial fisheries, especially juveniles with total length of 10–15 cm [4]. Recently, this fishery resource was highly overfished for human consumption resulting in its decline compared with historical fishery resource data [4]. Furthermore, due to high market demand and ecological value, the culture of *K. punctatus* have developed rapidly in the past decades. However, various problems have appeared in intensive culture. For instance, the ingestion, growth and survival of *K. punctatus* often have been severely affected by the low temperature stresses, indicating that this species is adaptive to warm water. Meanwhile, some researchers suspected that *K. punctatus* has a tendency to migrate to brackish waters during reproduction stages and therefore the reproduction behavior might be associated with seawater salinity [4]. However, limited evidences were applied to discuss the relevant regulatory mechanism of *K. punctatus* at the genetic level.

Next-generation sequencing techniques such as RNA-seq have been widely used in the gene expression profiling and pathway studies. RNA-seq has many advantages compared with the microarray technology, which needs drafting genome sequence or EST information [5,6]. RNA-seq technology allows simultaneous analyses of all of the processes that are regulated at the transcription level, including metabolism, protein homeostasis and other regulatory cellular processes [7,8]. It has provided the opportunity to investigate subtle changes in regulatory sequences contributing to gene expression divergence [9,10]. Additionally, RNA-seq also provides an opportunity to enable systematists the ability for phylogenetic analysis of fishes based on hundreds to thousands of loci [11]. However, successful examples only have been applied in 11 Clupeiformes fishes, including *Alosa alosa* [12], *Alosa pseudoharengus* [13], *Sardina pilchardus* [14], *Coilia nasus*, *Engraulis encrasicolus*, *Clupea harengus*, *C. pallasii*, *Brevoortia tyrannus*, *Tenualosa ilisha*, *Denticeps clupeoides* and *Amblygaster clupeoides* [15]. Therefore, we considered that the transcriptome data are very valuable for future research on the phylogenetic position and regulatory mechanism of *K. punctatus*.

More complete genetic information from genomic or transcriptomic data based on next-generation sequencing techniques can help us accurately explain the evolutionary biology of *K. punctatus*. Considering the lack of the whole-genome information, the present study first sequenced and *de novo* assembled the whole tissue transcriptome of *K. punctatus*. Furthermore, we combined genomic sequences of other fishes to obtain the single-copy orthologous genes shared by all research species. These single-copy orthologous genes are very conserved and inherited in the evolutionary processes of *K. punctatus*, and are closely related to the basic life activities, which will help us to explore the adaptive evolution mechanism of *K. punctatus*.

## Materials and methods

### Sample collection, RNA extraction and Illumina sequencing

Five healthy *K. punctatus* were obtained from an aquaculture farm in Zhoushan (China) on 26 July 2017. These *K. punctatus*s were quickly transported to the Laboratory of the School of Fishery at Zhejiang Ocean University (Zhoushan, China) and then were anesthetized using tricaine methanesulfonate (100 mg/l) to death. Then, muscle, liver, gill, heart, kidney, swim bladder and sexual gland of each individual were rapidly sampled, snap-frozen in liquid nitrogen and stored at −80°C for the RNA extraction. Total RNA of every tissues of five fishes were extracted, using a standard *TRIzol Reagent Kit* and following the manufacturer’s protocol, and then the RNA was pooled in equal amounts to ensure complete transcriptome information. The quantitative evaluation of total RNA was done by using the Qubit 2.0 Fluorometer (Invitrogen; Q32866). We then purified mRNA by depleted rRNA from total RNA using the RNA Purification Beads. First-strand cDNA synthesis was performed by combining 17 μl of fragmented mRNA with 6 μl of *First Strand Buffer* and 2 μl of *Strand Enzyme Mix*, and incubated at 25°C for 10 min, 42°C for 15 min and 70°C for 15 min. For second strand synthesis, we added 20 μl of *Second Strand Buffer* and 5 μl of *Strand Enzyme Mix* into 25 μl of *First Strand cDNA*, and incubated at 16°C for 60 min. Then, double-stranded cDNA was purified by using 90μl of *VAHTS™ DNA Clean Beads* (1.8×). Subsequently, *End Prep Mix*, *dA-Tailing Buffer Mix* and *RNA Adapter* were applied to end repair, A-tailing and adapter ligation of the double-stranded cDNA, respectively. The linked product was purified by using 40 μl of *VAHTS™ DNA Clean Beads* (1×) and the fragment size was selected by using 80 μl of *VAHTS™ DNA Clean Beads* (0.7×). For the cDNA fragments enrichment reaction, we added 5 μl of *PCR Primer Mix* and 25 μl of *Amplification Mix 1* into 20 μl of purified product and ran the initial denaturation at 98°C for 30 s, followed by 15 cycles of denaturation at 98°C for 10 s, annealing at 60°C for 30 s, and extension at 72°C for 30 s, and finished with a final extension at 72°C for 5 min. RNA-sequencing libraries were quantified using 8% polyacrylamide gel. Then the library was sequenced on the Illumina HiSeq™ 2500 platform and 150-bp paired-end reads were generated.

### Transcriptome *de novo* assembly and annotation

All raw reads in FASTQ format quality control relied on FastQC 0.11.2, clean reads were obtained by removing reads with sequencing adaptors, unknown nucleotides (N ratio > 10%) and low quality (quality scores < 20) based on Trimmomatic 0.36 [16]. Furthermore, 10000 clean reads were randomly selected and compared with nucleotide sequences (NT) database to examine the possible contamination [17]. Contamination detection was produced using blastn with the following parameters: evalue ≤ 1e-10, similarity > 90% and coverage > 80%. The remaining high-quality clean reads were *de novo* assembled using Trinity 2.4.0 [18] with the parameter: min_kmer_cov 2. Then the redundant transcripts were removed and further spliced the longest unigenes. Additionally, all clean reads then be aligned to transcripts and RSeQC 2.6.1 [19] was applied to analyze the distribution frequency of redundant reads. Homology searches were acquired by comparing all unigenes against the cluster of orthologous groups of proteins (COG), eukaryotic ortholog groups (KOG), non-redundant protein sequences (NR), protein families (PFAM), swiss-prot protein sequence (Swissprot), translation from EMBL (TrEMBL) and NT databases using the National Center for Biotechnology Information (NCBI) Blast+ (E-value < 0.00001; [20]). Then, Blast2GO software produces gene ontology (GO) annotations of unigenes on the basis of protein annotation results of Swissprot and TrEMBL databases [21]. Furthermore, the biochemical metabolic pathways and function of gene product were predicted based on Kyoto Encyclopedia of Genes and Genomes (KEGG) pathway annotation using KAAS (KEGG Automatic Annotation Server; http://www.genome.jp/kaas-bin/kaas_main; [22]). Additionally, BUSCO software was applied to the quantitative assessment of the completeness of non-redundant sequences [23].

### Predict the gene structure

In the present study, we identified coding sequences (CDSs) of unigenes by comparing all unigenes with the protein databases using blastx (E-value < 0.00001) and sequentially followed a fixed order of NR, Swissprot and TrEMBL databases. Then, TransDecoder 3.0.1 was used to predict the CDS of unigenes that not matched in the Swissprot and TrEMBL databases. We further analyzed the simple sequence repeats (SSRs) of all unigenes using MISA 1.0. SSRs were identified according to the repeats of Mono-nucleotide (1 bp), Di-nucleotide (2 bp), Tri-nucleotide (3 bp), Quaed-nucleotide (4 bp), Penta-nucleotide (5 bp) and Hexa-nucleotide (6 bp) was 10, 6, 5, 5, 5 and 5, respectively.

### Sequencing saturation analysis

To testing the gene quantification accuracy under different percentages of total sequencing data, saturation analysis was performed in the present study. The saturation curve algorithm is as follows: firstly, we separately conducted a gene quantitative analysis for 10, 20, 30, …, 90% of total sequencing data, the gene expression levels at different percentages as final numerical values (FNVs). Then, each gene quantification accuracy at different percentages was evaluated by comparing transcripts per million (TPMs) and FNVs, and the difference of less than 15% was used as the filtering thresholds [24].

### Orthologous genes determination and alignment

To investigate the orthologous genes shared by *K. punctatus* and other fishes (closely and distantly related fishes), we performed an extensive orthologous gene comparison among eight fishes, including three Clupeiformes fishes (*A*. *alosa*, *A*. *pseudoharengus*, and *S. pilchardus*), one Characiformes fish (*Astyanax mexicanus*), one Lepisosteiformes fish (*Lepisosteus oculatus*), one Beloniformes fish (*Oryzias latipes*), one Perciformes fish (*Dicentrarchus labrax*), and one Gadiformes fish (*Gadus morhua*). Firstly, we obtained the unigene sequences of the above eight fishes from the NCBI database under the accession number GETY00000000, GFCK00000000, GGSC00000000, GFIE00000000, GFIM00000000, GFIO00000000, GFJW00000000 and GFIX00000000, respectively. The longest ORFs of each transcriptome data were extracted for orthologous gene analyses using TransDecoder 3.0.1 with the parameter: m 200. Then, the single-copy orthologous genes among all species were extracted by OrthoMCL [25]. All single-copy orthologous genes of each species were aligned and linked a single protein sequences based on the mafft program [26]. Conserved sequences were extracted from each protein sequences using Gblocks with parameter: −t = p [27]. We further calculated the optimal amino acid substitution model using ProtTest. Finally, multiple sequence alignment results were used to construct the phylogenetic tree from the super-alignment of the CDSs using RAxML (randomized axelerated maximum likelihood) software. FigTree was applied to draw the phylogenetic tree [28]. The divergence time was estimated using the r8s software and a molecular clock data from the divergence time between *O. latipes* and *D. labrax* (105–154 MYA) from the TimeTree database [29]. Additionally, HMMER (biosequence analysis using profile hidden Markov models) software [30] was also applied to search the single-copy orthologous genes among all species based on the single-copy conserved actinopterygii orthologs database (https://busco.ezlab.org/datasets/actinopterygii_odb9.tar.gz). All single-copy orthologous genes of each species were then applied to the study of phylogenetic construction based on the same method as above.

## Results

### Transcriptome sequencing and reads assembly

**Figure 1:**
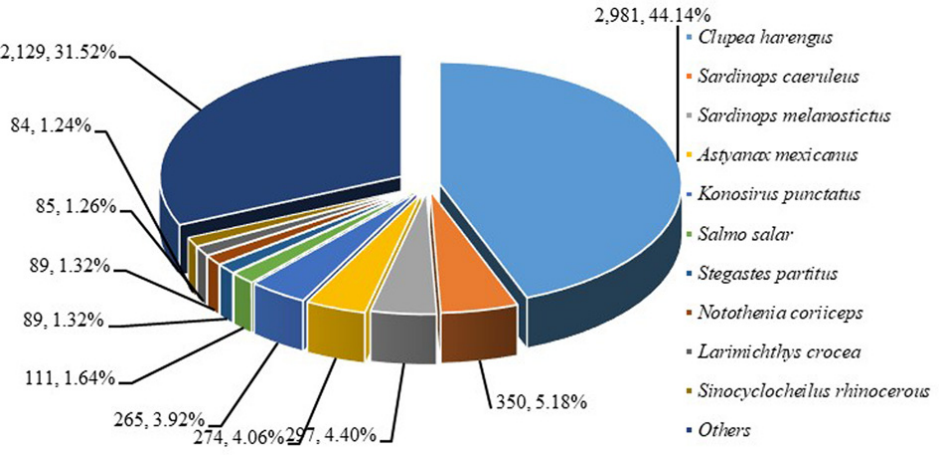
The top ten species of 10000 clean reads mapped in NT database

After cleaning and quality testing, a total of 46087110 clean reads were screened out from 47454642 raw reads, corresponding to 6531521430 nucleotides. The average read length, Q30 bases ratio, N bases ratio and GC bases ratio were 141.72 bp, 97.08, 0.00 and 54.57%, respectively. Additionally, 10000 clean reads were randomly selected and compared with NT database to examine the possible contamination, and 6754 clean reads had significant matches with sequences in the NT database. Results showed that a large proportion of clean reads were distributed among some species that were closely relative with *K. punctatus*, indicating no-genomic pollution (Figure 1).

**Figure 2:**
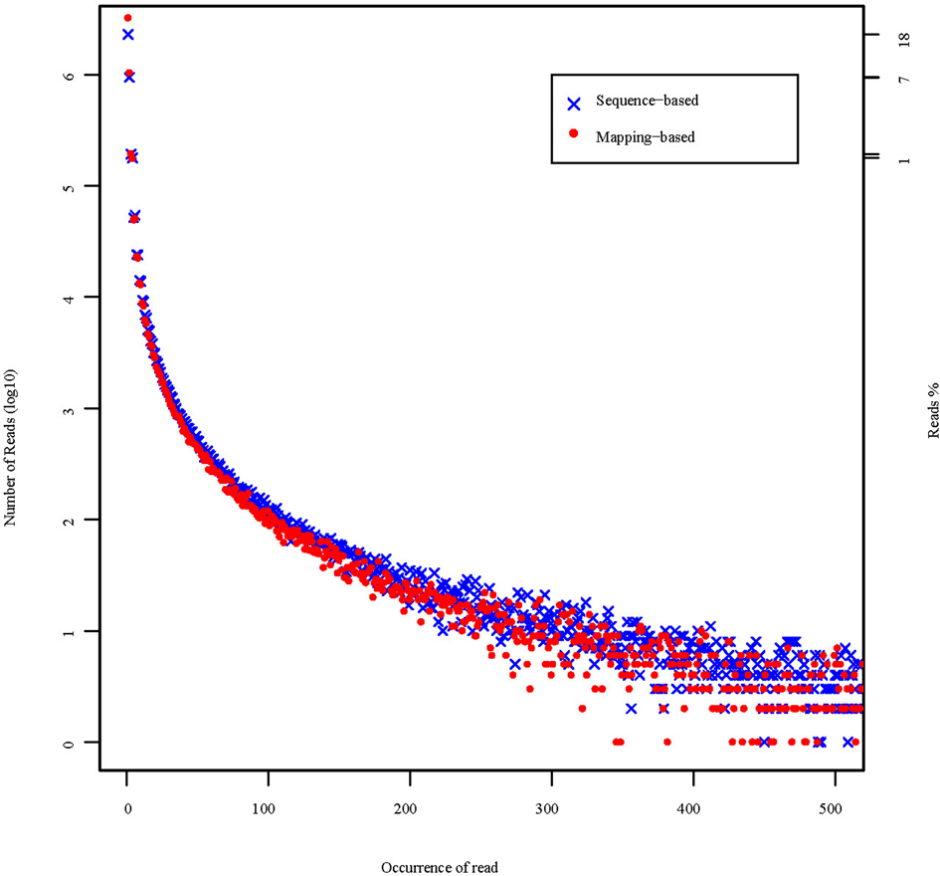
The distribution frequency of redundant reads

**Figure 3:**
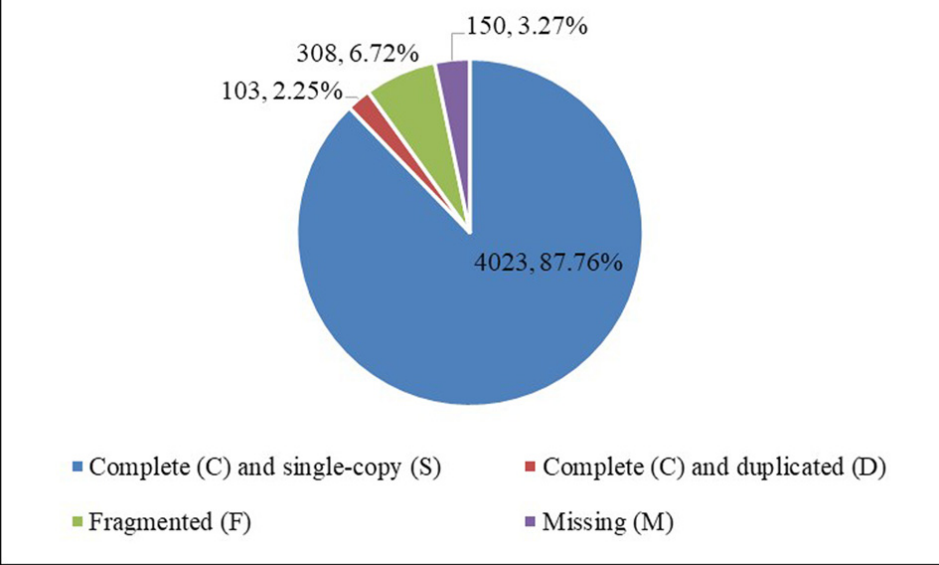
BUSCO assessment results

All high-quality clean reads were assembled to produce 122587 transcripts with an N50 of 1130 bp. Then the redundant transcripts were removed and further obtained 71610 longest unigenes with an N50 of 906 bp (Table 1). Furthermore, the number of redundant reads was reduced with increased frequency of redundant reads (Figure 2), and there was no peak in distribution curve. Therefore, we suspected that the content of redundant reads is normal. Additionally, 90.01% complete BUSCOs in the actinopterygii_odb9 database were covered by unigenes and therefore the completeness of *K. punctatus* transcriptome was be confirmed (Figure 3).

**Table 1:** Statistics for the assembly of *K. punctatus* whole transcriptome

	Transcripts	Unigenes
Number	122587	71610
≥500 bp	50871	24170
≥1000 bp	25175	11024
N50 (bp)	1130	906
N90 (bp)	284	255
Max length (bp)	17500	17500
Min length (bp)	201	201
Total length (bp)	87195434	43644528
Average length (bp)	711	609

### Gene annotation and functional classifications

**Figure 4:**
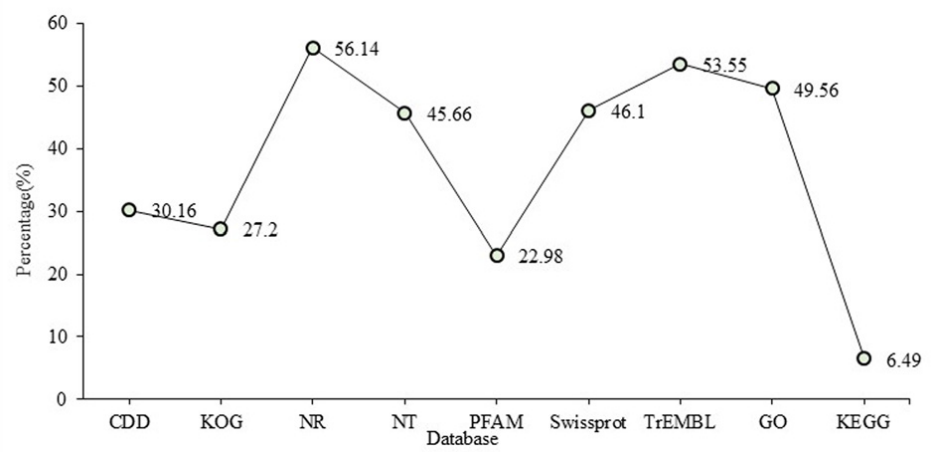
The annotation ratio of unigenes in each database

All unigenes were applied to analyze the GO and orthologous classifications based on protein databases. Results showed that a total of 43974 (61.41%) unigenes were annotated in at least on database and 3172 unigenes were annotated in all databases. Of all annotated unigenes, 21597, 19479, 40144, 32700, 16453, 33010, 38347, 35488 and 4646 unigenes had significant matches with sequences in the COG, KOG, NR, NT, PFAM, Swissprot, TrEMBL, GO and KEGG databases, respectively. The annotation ratio of each database is shown in Figure 4.

**Figure 5:**
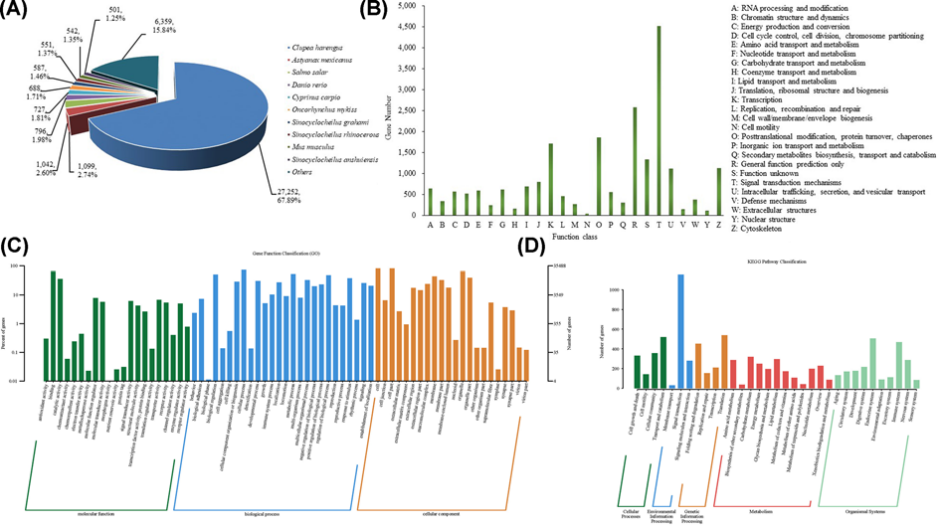
The annotation information of all unigenes blasted in protein database (**A**) The description information of similarity of unigenes comparison with these genes in Nr database. (**B**) KOG classification of putative proteins. (**C**) GO classification of all unigenes. (**D**) The significant KEGG classifications in the *K. punctatus* transcriptome.

We evaluated the approximate situation of *K. punctatus* and similar species, and obtained the functional information of homologous sequence by comparing all unigenes with the NR protein database. Results showed that 27252 (67.89%), 1099 (2.74%), 1042 (2.60%), 796 (1.98%), 727 (1.81%), 688 (1.71%), 587 (1.46%), 551 (1.37%), 542 (1.35%) and 501 (1.25%) unigenes were matched with genes from *Clupea harengus*, *Astyanax mexicanus*, *Salmo salar*, *Danio rerio*, *Cyprinus carpio*, *Oncorhynchus mykiss*, *Sinocyclocheilus grahami*, *Sinocyclocheilus rhinocerous*, *Mus musculus* and *S. anshuiensis*, respectively. The remaining unigenes (6359, 15.84%) were hits in the other species (Figure 5A).

KOG databases included families of both known and unknown 3D structure, covering the protein sequence space more completely. In the present study, KOG classification resulted in 19479 unigenes categorized into 25 categories (Figure 5B). Among these categories, the first three largest groups were T category (4527 unigenes), R category (2591 unigenes) and O category (1870 unigenes), representing ‘Signal transduction mechanisms’, ‘General function prediction only’ and ‘Post-translational modification, protein turnover, chaperones’, respectively.

As an international standardized gene function classification system, GO classification can provide a dynamically updated controlled vocabulary and can exactly define gene characteristics. GO classification analysis was carried out in the present study and a total of 35488 unigenes were successfully mapped to existing gene categories (Figure 5C). Among these functional groups, the terms of ‘cell’, ‘binding’ and ‘cellular process’ were dominant in ‘cellular component’, ‘molecular function’ and ‘biological process’, respectively.

In addition, KEGG database analysis was used to understand high-level functions and utilities of the biological system in the present study. As a result, a total of 4646 unigenes were assigned to 48 KEGG pathways (Figure 5D). Results provided a new insight into the adaptive mechanism of *K. punctatus* and indicated that first three largest groups KEGG classifications were ‘Signal transduction’ (1155 unigenes), ‘Translation’ (539 unigenes) and ‘Transport and catabolism’ (519 unigenes).

### Gene structure analysis

**Figure 6:**
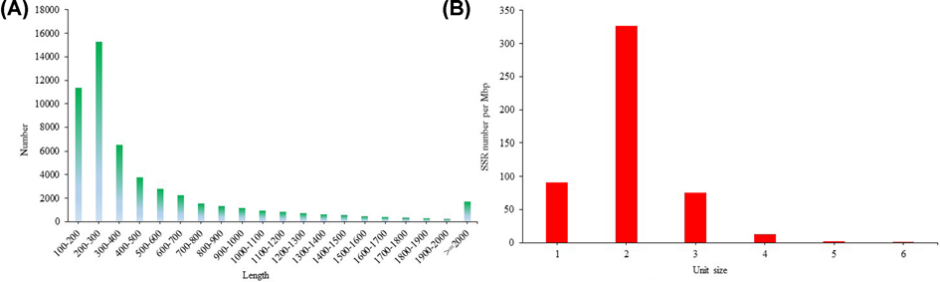
Gene structure analysis results (**A**) The length distribution of the nucleotide sequences of CDS. (**B**) The density distribution of SSRs.

In the present study, all unigenes were further analyzed to predict the gene structure. We have obtained a total of 54864 nucleotide sequences and protein sequences of coding regions. Among them, 53053 nucleotide sequences were longer than 100 bp. The length distribution of the nucleotide sequences of CDS was shown in Figure 6A. Additionally, we identified 17326 SSRs based on the screening criteria and the density distribution of SSRs was shown in Figure 6B.

### Sample sequencing saturation and sample gene saturation

**Figure 7:**
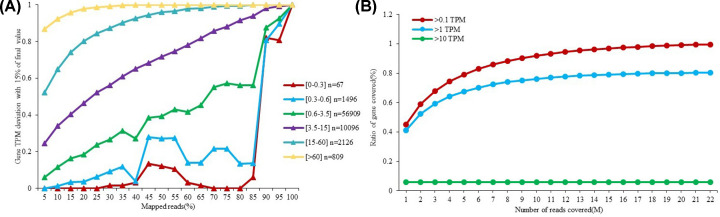
Sequencing saturation analyses (**A**) Sample sequencing saturation curve: x-axis is the percentage of mapped reads, and y-axis is the percentage of genes that is quantitative error is less 15%. Different colors represented different saturation curve of genes with different expression levels. (**B**) Sample gene saturation curve: x-axis is the number of clean reads, y-axis is the percentage of identified genes. Different colors represented different expression thresholds.

The detection effective of transcripts were closely related to sequencing data volume, due to different genes of various expression levels. Saturation analysis can apply to evaluate the accuracy of gene expression under different sequencing amount. Therefore, analysis of sample sequencing saturation and sample gene saturation were designed in the present study, and the saturation curves were shown in Figure 7A,B. Results showed the sequencing saturation curve of high expression genes faster entry into the platform phase. Additionally, the number of identified genes slowly increased to a saturated value with the growth of data. Therefore, we hypothesized that the detection of gene expression might be effective.

### Phylogenetic analysis for *K. punctatus* and other fishes

**Figure 8:**
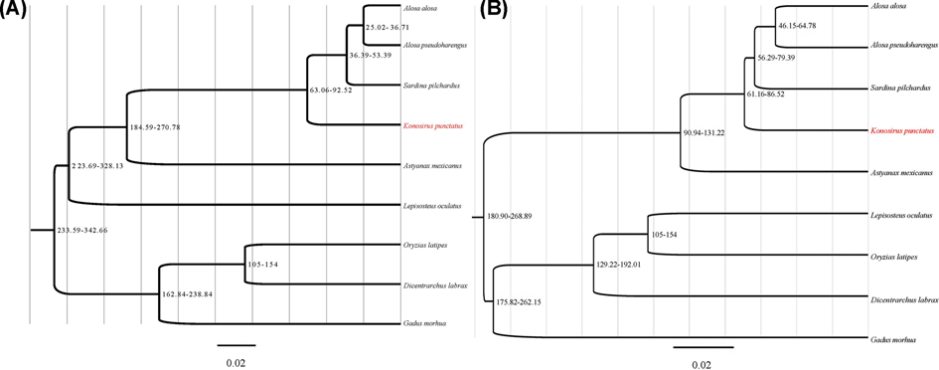
The phylogenetic tree based on single-copy orthologous genes (A) Single-copy orthologous genes obtained by OrthoMCL software. (B) single-copy orthologous genes obtained by HMMER software The phylogenetic tree based on single-copy orthologous genes obtained by OrthoMCL (**A**) and HMMER (**B**) method, respectively.

In the present study, two softwares were applied to explore the single-copy orthologous genes of *K. punctatus* and other fishes. Based on the OrthoMCL software, we identified a set of 117 single-copy orthologous genes longer than 200 bp among nine fishes. Then the concatenated alignment of genes produced a data matrix with 25172 bp amino acids. However, 3120 single-copy orthologous genes were obtained using HMMER software in Actinopterygii orthologs database and the concatenated alignment of genes produced a data matrix with 797332 bp amino acids. Furthermore, the maximum-likelihood method implemented in the RAxML package with the JTT+I+F model were used to construct the phylogenetic tree from the data matrix of the conserved exons (Figure 8). Unsurprisingly, the constructed phylogenetic structure based on OrthoMCL software and HMMER software indicated that *K. punctatus* were clustered together with *A. alosa*, *A. pseudoharengus* and *S. pilchardus*, and they all belonged to the Clupeidae. Meanwhile, the differentiation time showed that *K. punctatus* diverged from the common ancestor with *A. alosa*, *A. pseudoharengus* and *S. pilchardus* at approx. 61.16–92.52 MYA (Figure 8).

## Discussion

In the present study, we first conducted RNA-seq to capture significant portion information of the *K. punctatus* transcriptome data for further apply to construct the phylogenetic relationship of *K. punctatus*. Finally, the results provided a novel understanding for the identification of function genes and reconstitution of phylogenetic relationships of *K. punctatus*.

### Sequencing and *de novo* assembly of the *K. punctatus* transcriptome

Thus far, the genomic data of *K. punctatus* has not been sequenced. In this context, whole-tissue trancriptomic sequencing obtained in the present study will contribute to the development of genome-wide genetic information of *K. punctatus*. After filtering, a total of 46087110 clean reads were applied to the *de novo* assembly of *K. punctatus* transcriptome. Results showed that the unigene sequences obtained after assembly and removal of redundancy have relatively lower average length (609 bp) and N50 value (906 bp). The average length and N50 value of *K. punctatus* transcriptome sequence were lower than those (average length of 1520 bp and N50 of 2319) of *C. nasus* also belonging to the Clupeiformes [31]. However, the annotation efficiency (61.41%) of *K. punctatus* transcriptome sequence was higher than that (56.80%) of *C. nasus*. We suspected that the absence of reference genome affects the assembly and annotation efficiency of transcriptomic sequences. Meanwhile, repeated and highly similar sequences in vertebrate (including teleost) genomes can cause splicing collapse during *de novo* assembly. In fact, the algorithm of the *de novo* assembly strategy only completed the construction of the transcripts with high expression but ignored the transcripts with low expression [32]. In conclusion, we do not deny that the lack of whole genome and the selection of sequencing strategies have affected the integrity of the *K. punctatus* transcriptome. Even so, we still believe that these data expand the currently available genomic resources for *K. punctatus*. It is worth noting that multitissue mixing in the present study may leads to the excessive ribosomal RNA content during sequencing process. However, we continue to believe that the integrity of sequencing information is positively correlated with the amount of tissues used. In fact, multiple elution of RNA during library construction also can effectively reduce the content of ribosomal RNA.

Based on the *K. punctatus* transcriptome sequence, the total number of identified SSRs was 17326. According to the density distribution of SSRs, the dinucleotide repeats had the highest density, which was similar to other fishes, such as *Pseudosciaena crocea* [33] and *Megalobrama amblycephala* [34]. Meanwhile, the density distribution of SSRs decreases with the length of the repetitions, because long mutations have high mutation rates [35,36].

### Phylogenetic status of *K. punctatus* based on single-copy orthologous genes

Single-copy orthologous genes were housekeeping genes, which were very conserved and inherited lineally in the evolution of species. The differentiation of orthologous genes directly lead to the speciation [37]. Meanwhile, the use of multiple single-copy orthologous genes can also avoid conflicting gene trees caused by different genetic markers [38]. Another pivotal application of single-copy orthologous genes was the accurate prediction of biological differentiation time [39]. This is the case because the molecular clock based on multiple single-copy orthologous genes can eliminate the estimation errors caused by differences in the evolutionary rate [39]. Therefore, we can more accurately determine the phylogenetic status and differentiation time of *K. punctatus* based on hundreds to thousands of single-copy orthologous genes.

In the present study, the number of single-copy orthologous genes obtained by two softwares was significantly different. We hypothesized that software design principles and data integrity influenced the final number of single-copy orthologous genes. This also means that more complete single-copy orthologous genes can be obtained using genomic data, but the application of genomic data in phylogenetic studies is challenged by sequencing costs and computational methods [40]. However, it is undeniable that transcriptome data are still an effective means to obtain single-copy orthologous genes in the absence of genomic data [41]. It is worth noting that the number of single-copy orthologous genes obtained by OrthoMCL software is far less than that obtained by HMMER software. This is probably because OrthoMCL software can screen the orthologous genes more rigorously by Blast pairwise comparison [3]. Meanwhile, two whole-genome duplications (WGDs) events in the genome evolution of teleost produced a large number of duplicate genes, which may also affect the exploitation of single-copy orthologous genes [42,43]. However, it is not clear which software is better suited for single-copy orthologous gene analysis.

Although the number of single-copy orthologous genes used in the present study is different, the structure of constructed phylogenetic tree is similar. Unsurprisingly, the constructed phylogenetic trees based on single-copy orthologous genes (117 or 3120) were significantly congruent with the prevailing morphological and molecular biological view of Clupeiformes [44]. In other words, fishes from the Clupeiformes eventually cluster into one branch. There is no denying that inadequate whole-genome genetic information on Clupeiformes still limits our accurately determination of phylogenetic status of *K. punctatus*. Therefore, subsequent research needs to continue to develop whole-genome genetic information of more closely or distantly related species of *K. punctatus*. Results also showed that the differentiation time based on the OrthoMCL software and HMMER software were not completely consistent. This is probably because the number of single-copy orthologous genes obtained based on the two softwares were different, which ultimately leads to the difference in differentiation time. Previous researches have confirmed that although the molecular clock hypothesis can accurately predict the genetic differentiation time of species, the stability of molecular clocks is still affected by genetic evolution [39]. In fact, our previous research on *Sillago* species also showed that the number of single-copy orthologous genes may influence the differentiation time of species [41]. However, whether the differentiation time of *K. punctatus* based on the single-copy orthologous genes generated by HMMER software was more accurate still needs further verification.

In brief, our research confirms that transcriptome data can be applied to explore large amount of single-copy orthologous genes and further to construct accurate phylogenetic relationships. It is worth noting that the single-copy orthologous genes based on transcriptome data may be incomplete. Therefore, it is necessary to complete the sequencing of the species genome. Additionally, under the premise of guaranteeing the quality of single-copy orthologous genes, the accuracy of phylogenetic relationships may be positively correlated with the number of single-copy orthologous genes used.

## Conclusion

Since the development of next-generation sequencing technology, RNA-seq has been widely used as an efficient and accessible approach to obtain the expression information in organism with no reference genome. Here, we sequenced and assembled the transcriptome of *K. punctatus* and this was the first systematic report of its whole transcriptome. Additionally, we obtained 117 and 3120 single-copy orthologous genes based on OrthoMCL and HMMER method, respectively. Then, we constructed the phylogenetic relationship of *K. punctatus* based on single-copy orthologous genes and results showed that *K. punctatus* diverged from the common ancestor with *A. alosa*, *A. pseudoharengus* and *S. pilchardus* at approx. 6–92.52 MYA. As supplementary information on the molecular aspects, our results presented here provided a basic resource for the phylogenetic analysis and regulatory mechanism of *K. punctatus*. This information will play critical roles for future understands the genomic level of *K. punctatus*.

## Data Availability

All research data are included in the main text of the manuscript. The raw reads of the present study were uploaded to the SRA databases of NCBI under BioProject number PRJNA525541, with accession number SRR8668068 (https://www.ncbi.nlm.nih.gov/sra/?term=SRR8668068). The assembled and annotated transcript has been deposited at DDBJ/EMBL/GenBank under the accession number GHHF00000000 (https://www.ncbi.nlm.nih.gov/nuccore/GHHF00000000). The version described in the present paper is the first version, GHHF01000000.

## Abbreviations

CDS: coding sequence
COG: cluster of orthologous groups of proteins
FNV: final numerical value
GO: gene ontology
KEGG: Kyoto Encyclopedia of Genes and Genomes
KOG: eukaryotic ortholog group
NCBI: National Center for Biotechnology Information
PFAM: protein family
RAxML: randomized axelerated maximum likelihood
SSR: simple sequence repeat
Swissprot: swiss-prot protein sequence
TrEMBL: translation from EMBL
